# A Review of Cyclodextrin Encapsulation and Intelligent Response for the Release of Curcumin

**DOI:** 10.3390/polym14245421

**Published:** 2022-12-11

**Authors:** Jing Li, Fang Xu, Yujie Dai, Jiawen Zhang, Yuan Shi, Danning Lai, Natthida Sriboonvorakul, Jiamiao Hu

**Affiliations:** 1 Engineering Research Centre of Fujian-Taiwan Special Marine Food Processing and Nutrition, Ministry of Education, Fuzhou 350002, China; 2 Department of Clinical Tropical Medicine, Faculty of Tropical Medicine, Mahidol University, Bangkok 73170, Thailand

**Keywords:** cyclodextrin, curcumin, supramolecular system, stimuli-responsive delivery

## Abstract

To overcome the low water solubility and low bioavailability of curcumin (CUR), multiple delivery strategies have been proposed. Among these, cyclodextrin-based carriers have been widely used for the encapsulation and delivery of CUR. Cyclodextrins (CDs), as natural oligosaccharides, have been well known for their biodegradability, biocompatibility, non-toxicity, and internal hydrophobic and external hydrophilic structural features. This paper summarizes the recently reported CD-based carriers for encapsulating CUR. Particularly, the polymerization properties of CD self-assembly to enhance the encapsulation of CUR are discussed. In addition, the current progress on stimuli-responsive CD carriers for controlled release of CUR is described, which laid an important foundation for the development of CUR-based precision therapy in clinical practice. In conclusion, this review may provide ideas for the future development of a CD-based encapsulant for CUR.

## 1. Introduction

Curcumin (CUR) is a natural yellow polyphenolic compound extracted mainly from the roots of some plants in Zingiberaceae (commonly known as the ginger family) and Araceae [1]. CUR has received wide attention because of its various biological activities, including antioxidant, anti-inflammatory, anti-bacterial, anti-viral, anti-cancer, anti-diabetic, and neuroprotective [2,3,4,5,6]. Currently, many studies have shown that it has good effects in the treatment of cancer, cardiovascular disease, inflammation, diabetes, and neurological disorders [7,8,9,10,11]. Furthermore, the safety of CUR has been confirmed by pharmacological and toxicological studies; even at doses of 8 g/day to 12 g/day, it does not cause significant toxic side effects to humans [12]. Therefore, the US Food and Drug Administration approved curcumin as “Generally Recognized as Safe” (GRAS) [13]. However, poor water solubility and stability are the main reasons for the failure of most phytochemicals [14]. CUR also suffers from these defects, being highly lipophilic and having very low intrinsic water solubility, with a solubility of 11 μg/mL in water [15]. In addition, CUR is also susceptible to degradation during storage due to environmental factors such as light, heat, and oxygen, which greatly limits its application in pharmaceuticals, food, and other related fields [16]. Furthermore, CUR is poorly absorbed in the intestinal tract and is rapidly metabolized in the liver, preventing it from being effective in the body [17]. Therefore, there is an urgent need to develop methods that not only improve its water solubility and stability but also regulate its biodistribution after administration.

Currently, the application of cyclodextrins for curcumin encapsulation and delivery is a promising strategy to overcome the aforementioned limitations. Cyclodextrins (CDs), obtained by enzymatic digestion of starch using cyclodextrin glycosyltransferase, are made from D-glucopyranose units connected by α-1,4-glycosidic bonds, usually containing six, seven, or eight glucose units, called α, β, and γ-CD, respectively [18]. Due to the internal hydrophobic and external hydrophilic properties, CDs can be loaded with various hydrophobic drugs by forming inclusion complexes, thus improving the water solubility of these drugs [19]. In addition, CDs are safe for humans, so they are considered to be ideal carriers for various drugs [20]. Furthermore, the latest studies further demonstrate that CD could be used to develop intelligent, stimulus-responsive drug carriers, which can be stimulated by factors including changes in pH, light, and enzymes to release the encapsulated drugs [21]. This strategy could achieve targeted and on-demand drug delivery at specific pathological sites and decrease the undesirable effects on the sensitive normal tissues, which has drawn great attention in the field of cancer therapy due to the differences between the microenvironment of normal and tumor cells [22]. Indeed, the stimulus-responsive CDs-based nanoparticles have also been used successfully to enhance the therapeutic potential of CUR [21]. Therefore, this review will summarize the literature related to CD-based carriers for encapsulating CUR as well as discuss the latest progress on the stimulus-responsive CD carriers for the precise release of CUR.

## 2. Curcumin–Cyclodextrin Supramolecular System

Supramolecular systems are based on molecular recognition, in which two or more molecules are bound by intermolecular non-covalent bonding forces to form complex and ordered entities or aggregates with specific functions and properties [23]. CD can be used to encapsulate CUR due to its cylinder structure, forming a relatively simple host–guest supramolecular system, which can improve the water solubility and bioavailability of CUR [24,25,26]. Notably, instead of being limited to a single host–guest form, CDs monomers could also be constructed into functional polymers by chemical modification or physical aggregation [27,28]. This allows the cyclodextrin polymer to achieve enhanced loading capacity and solubility for CUR [29]. Furthermore, introducing other functional molecule(s) into the curcumin–cyclodextrin supramolecular system was also proven to be a promising strategy for its delivery properties. Herein, different supramolecular systems of curcumin cyclodextrins are presented.

### 2.1. Curcumin–Cyclodextrin Supramolecular System with Cyclodextrin as the Carrier

#### 2.1.1. Natural Cyclodextrins

The first wave of studies has focused on the interaction of CUR with natural CDs. The researchers used solvent evaporation, freeze-drying, kneading, and other methods to prepare curcumin–cyclodextrin inclusion complexes [19]. When inclusion complexes are formed, the crystallinity, solubility, and optical properties of CUR molecules are changed, and the properties are subsequently measured using thermal analysis, spectroscopy, or chromatography to verify the formation of the inclusion complex [30]. The relevant articles on cyclodextrin-encapsulated curcumin are summarized in Table 1.

**Figure 1:**
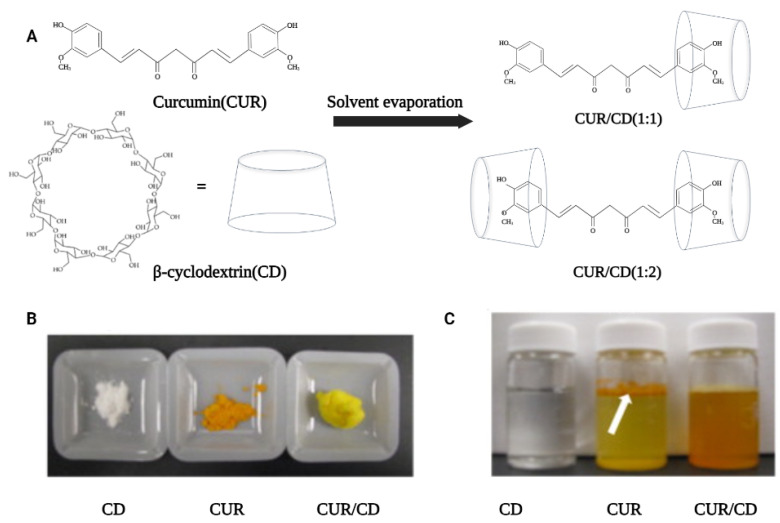
Preparation process and solid-liquid form of CUR/β-CD. (**A**) Schematic diagram of the preparation of curcumin/β-cyclodextrin supramolecules by solvent evaporation. (**B**) Solid powder samples of β-cyclodextrin, curcumin and curcumin/β–cyclodextrin inclusion complex (CUR/CD). (**C**) Aqueous solutions of CD, CUR and CUR/CD inclusion complex (5 mg/mL) [31].

Solvent evaporation is the most commonly used method for the preparation of curcumin–cyclodextrin complexes due to its simplicity (Figure 1A). For instance, this method was used by Yallapu et al. to prepare CUR/β-CD as a light-yellow fluffy powder with excellent aqueous solubility (Figure 1B). Unlike pure curcumin, which readily precipitates in an aqueous solution, curcumin encapsulated by β-CD showed an increased solubility up to 1.84 mg/mL in water (Figure 1C) [31]. In another study, López-Tobar et al. used β-CD and γ-CD as the carriers to encapsulate curcumin and analyzed the stability of the inclusion complexes using Raman spectroscopy. The results illustrated that H-bonds play an important role in the encapsulation process of curcumin, prompting changes in the structure of curcumin from the planar keto-enol tautomer to the non-planar diketone tautomer. These changes may cause an increase in the bioavailability, bioactivity, and chemical stability of curcumin. It is also interesting to note that the authors mention that γ-CD affords better encapsulation than β-CD, which may be due to the fact that the size matching between curcumin and gamma CD cavity is better [32]. Similarly, Alizadeh et al. also demonstrated that hydrogen bonds play a key role in enhancing the physiological activity of CUR [33]. Their study compared the antioxidant activity of free CUR with CUR/β- or γ-CD. The results indicated that CUR/γ-CD had superior antioxidant activity to that of CUR/β-CD or free CUR. This was attributed to the formation of one or more intermolecular hydrogen bonds upon the complexation of CUR by the CDs, which affected the intramolecular hydrogen bonds of CUR, thus enhancing the hydrogen-donating ability (enhanced antioxidant activity) of CUR molecules. In addition, Jahed et al. further investigated the interaction forces between CUR and CD using NMR spectroscopy [34]. The 1H NMR and 2D ROESY spectra confirmed that the chemical shifts of the internal protons of β-CD (H-3 and H-5) were shifted after encapsulation, and there was a cross-peak between the H-3 proton of β-CD and the aromatic rings group of CUR. These studies show that the driving forces involved in the CD encapsulation of CUR include hydrophobic interactions between host and guest, hydrogen bonding, van der Waals forces, and other non-covalent bonding forces. These driving forces sometimes act individually, but in most cases, multiple forces act synergistically to promote the formation of supramolecular systems.

#### 2.1.2. Cyclodextrin Derivatives

Natural CDs themselves have many shortcomings, such as the small pore size of α-CD cavities and relatively low water solubility of β-CD, which hinder the further application of CDs. For this reason, researchers have introduced modified groups to obtain cyclodextrin derivatives with different properties or functions, while keeping the basic skeleton of CD macrocycles unchanged. These derivatives are classified as hydrophilic, hydrophobic, ionic, and amphiphilic; and these modified CDs have also been widely investigated in the application of curcumin encapsulation.

Since the main objective of curcumin–cyclodextrin complexation is to obtain an inclusion complex with high water solubility, therefore, hydrophilic cyclodextrin derivatives are the primary choice for encapsulating CUR. For example, hydroxypropyl β-CD (HP-β-CD) is an alkylation product of β-CD. Alkylating the -OH groups on the periphery of β-CD with hydroxypropyl groups breaks the series of hydrogen bonds that these -OH groups make. This improves the solubility of the resulting HP-β-CD [35]. Li et al. used HP-β-CD as a carrier to improve the solubility and oral bioavailability of the poorly soluble drug CUR [36]. In rats, CUR/HP-β-CD and free CUR had similar pharmacokinetic behaviors after intravenous administration, and both had similar antitumor efficacy. Moreover, the oral bioavailability of CUR was enhanced 2.77-fold by HP-β-CD encapsulation. Additionally, Shityakov et al. demonstrated that the concentration of CUR in distilled water was about 60-fold higher when HP-γ-CD was used as compared to γ-CD due to the better hydrophilicity of HP-γ-CD [37]. These articles showed that CUR encapsulated in hydroxypropyl-modified CD resulted in a complex with superior water solubility. Articles that describe CUR encapsulated in other cyclodextrin derivatives are listed in Table 1.

**Figure 2:**
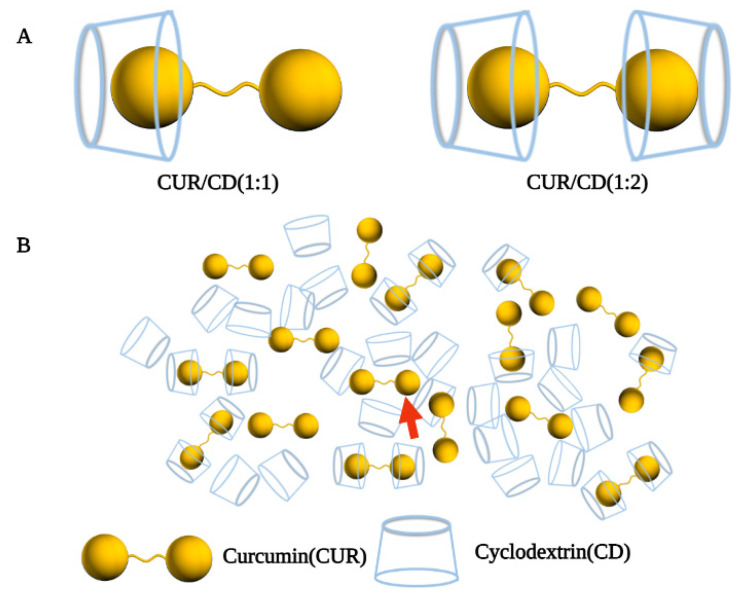
A mixed system. (**A**) 1:1 CUR/ HP-β-CD complex; 1:2 CUR/ HP-β-CD complex; (**B**) Mixed system of curcumin complexed cyclodextrin and the red arrow marks the CUR that was not in the inclusion complex [38].

Interestingly, Mai et al. prepared solid dispersions of CUR/HP-β-CD by grinding, freeze-drying, and common solvent evaporation methods [38]. The solubility of the inclusion complexes was increased 299, 180, and 489-fold, respectively, as compared with CUR crystals. Surprisingly, this solid dispersion did not consist of pure inclusion complexes but was rather a mixed system. The system consisted of free CUR molecules, inclusion complexes, CUR molecules not in inclusion complexes, and empty HP-β-CD molecules (Figure 2B). One or both of the aromatic rings of curcumin entered into the HP-β-CD cavity to form a 1:1 or 2:1 host-to-guest ratio inclusion complex (Figure 2A). In addition, unlike the free CUR molecules, the CUR molecules that were not in the inclusion complex may have been trapped in the cavities of a three-dimensional network structure formed by the polymerization of multiple cyclodextrin monomers. These results indicate that the actual process of curcumin inclusion in cyclodextrin does not present an ideal state in which the components are independent of each other, but rather is a complex system.

**Table 1:** Introduction to various curcumin–cyclodextrin supramolecular systems, with emphasis on cyclodextrin types, preparation techniques and structural characterization techniques.

CD Type	Preparation Method	Host to Guest Ratio	Characterization Techniques	Key Findings	Reference
HP-α-CD, HP-β-CD, HP-γ-CD	Co-evaporation, Freeze-drying	2:1, 1:1	FTIR, Raman spectra, XRD, UV-Vis and DSC	Raman spectroscopy can be used as an effective means of verifying the formation of inclusion compounds.	[39]
β-CD	Freeze-drying	-	FTIR, ^1^H NMR, DSC, TGA, XRD, SEM and TEM	CD enhanced the delivery of CUR in prostate cancer cells and improved its therapeutic efficacy compared to free CUR.	[31]
β-CD	Co-precipitation, Freeze-drying, Solvent evaporation	2:1	FTIR, Raman spectroscopy and XRD	The application of CUR/CD complex in vanilla ice creams intensified the color of the products and produced a great sensorial acceptance.	[40]
β-CD, γ-CD, HPβCD, 2-O-methyl-β-CD, HP-γ-CD	-	-	^1^H NMR, ^13^C NMR, FTIR and DSC	All five CUR/CD inclusions showed improved hydrolytic stability compared to CUR, but all had reduced antioxidant potential.	[41]
β-CD	Solvent evaporation, Freeze-drying	1:1	NMR,	NMR spectroscopy elucidates the mechanism by which β-CD enhances the water solubility of CUR.	[34]
β-CD, γ-CD	Solvent evaporation, Freeze-drying	1:1, 2:1, 4:1, 8:1	Raman spectroscopy and UV-Vis	Raman spectroscopy elucidates the mechanism of interaction between CUR and CD.	[32]
β-CD	Kneading, Co-precipitation	2:1	FTIR, SEM, XRD and UV-Vis	β-CD proven to be an excellent sustained release carrier for CUR.	[42]
β-CD	Saturated aqueous solution	-	FTIR and UV-Vis	CD may be used as a carrier to improve the release and therapeutic efficacy of CUR in lung cancer.	[43]
β-CD, γ-CD	The soluble method	1:1	UV-Vis, FTIR and ^1^H NMR	The CUR/CD inclusion has more antioxidant activity than free CUR.	[33]
β-CD	Kneading	1:1	NMR, FTIR, XRD, TGA and SEM	β-CD as a carrier enhances the anti-proliferative effect of CUR during the complexation process.	[44]
β-CD	Coprecipitation, Kneading, Simple mixing	2:1	DSC, TGA and ^1^H NMR	CUR/β-CD has greater color development than pure colorants and the use of the complexes in dairy products can produce a great sensorial acceptance.	[45]
HP-β-CD, Sulfobutylether-β-CD(SBE-β-CD)	Solvent evaporation, Freeze-drying, Autoclaving	-	^1^H NMR, Raman spectroscopy, DSC and XRD	The autoclaving method for complex formation was found to be the most efficient in terms of processing time and CUR encapsulation efficiency.	[46]
HP-β-CD	Solvent evaporation, Freeze-drying, PH shift	1:1	DSC and FTIR	Among the three methods of inclusion preparation, solvent evaporation is the most suitable method for preparation of CUR/HP-β-CD inclusion.	[47]
HP-β-CD	-	-	DSC	The PH value plays an important role in the formation of inclusion compounds.	[48]
HP-β-CD	Co-precipitation	-	FTIR, XRD and SEM	CUR/HP-β-CD inclusions have better potential than CUR nanoparticles for application in Alzheimer’s disease.	[49]
HP-β-CD	The grinding method	1:1, 2:1, 3:1	FTIR and DSC	CUR/ HP-β-CD in situ hydrogel are a promising formulation for melanoma treatment.	[50]
HP-β-CD	Kneading	1:1	SEM, DSC and FTIR	HP-β-CD complexation improves intestinal absorption of CUR.	[51]
HP-β-CD	Cosolvent-lyophilization	3:1	FTIR, XRD and DSC	The oral bioavailability of CUR was enhanced to 2.77-fold by the HP-β-CD.	[36]
HP-β-CD	Co-evaporation	1.35:1	UV-Vis, FTIR, NMR, XRD, DSC, TGA and SEM	A supramolecular system for the complexation of the modified CUR with HP-β-CD was established.	[52]
HP-β-CD	Grinding, Freeze-Drying, Common solvent evaporation	-	XRD, FTIR and DSC	The solid dispersion system consisting of CUR and HPβCD significantly increased the solubility of the drug compared to the inclusion complex.	[38]
SBE-β-CD	Freeze-drying, Kneading, Co-evaporation	1:1	^1^H NMR, FTIR, DSC and SEM	The CUR/SBE-β-CD complex has potential in the treatment of lung cancer.	[53]
Methyl-β-CD (M-β-CD)	Solvent evaporation	-	SEM	The CUR/M-β-CD inclusion complex showed higher antimicrobial potency than CUR nanoparticles.	[54]
Randomly methylated-β-CD (RM-β-CD)	Saturated aqueous solution	-	UV-Vis and FTIR	CUR forms a 1:1 inclusion complex with RM-β-CD.	[55]
Succinic acid-β-CD	-	-	-	Succinic acid-β-cyclodextrin affects the biological accessibility of curcumin in the circulation by modulating the binding of curcumin to bovine serum proteins.	[56]
γ-CD, HP-γ-CD	-	-	UV-Vis	HP-γ -CD has a better solubilizing effect on CUR than γ-CD.	[37]

### 2.2. Curcumin–Cyclodextrin Supramolecular System with Cyclodextrin Polymer as the Carrier

The structure of CD allows the formation of polymers with different structural characteristics, either covalently or non-covalently bonded [57]. These polymers have both the inclusion properties of CD and the favorable properties of polymers and are often used to form complexes with other molecules [58]. Indeed, several types of CD polymers have been reported to successfully encapsulate curcumin, which are summarized in Table 2.

#### 2.2.1. Cyclodextrin Self-Assembled Supramolecular Networks with Curcumin Encapsulated

Self-assembly can be defined as the process by which molecules or other assembled substrates spontaneously form ordered structural bodies through weak interactions [59]. CDs form amorphous, micelle-like, high-molecular-weight polymers by self-assembly to form a supramolecular system with the guest [27]. In this system, changes to molecular structure translate to differences in supramolecular forms, including superlattice crystals, micelles, vesicles, and liquid crystals. Each of these has unique structural features and new physicochemical properties completely different from those of the original constituent molecules [60].

**Figure 3:**
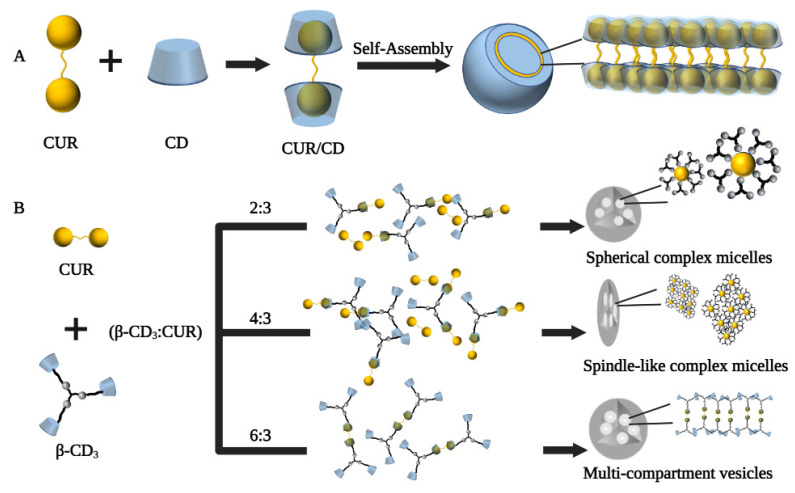
Preparation process of cyclodextrin self-assembled supramolecular networks. (**A**) Schematic diagram of the proposed mechanism of vesicle formation from CD and CUR [62]. (**B**) Schematic diagram of supramolecular self-assembly of three different shapes of curcumin/β-cyclodextrin trimers [63].

Supramolecular vesicles are hollow spheres with hydrophobic membranes and hydrophilic interiors, and the strategy of loading CUR in CD self-assembled supramolecular vesicles may be a good solution [61]. In a study by Ma et al., CD molecules encapsulated CUR through host–guest recognition to form a supramolecular amphiphile, which further self-assembled into vesicles due to hydrophobic interactions (Figure 3A). The resulting CUR/CD vesicles were hollow spheres with diameters in the range of 70–130 nm based on TEM and SEM observations, which increased the water solubility of CUR by 7000-fold (solubility up to 2 × 10^−4^ mol/L) [62]. In another study by Bai et al., the β-CD trimer(β-CD_3_) could form micelles in the presence of CUR as a guest unit by host–guest inclusion interaction and hydrophilic-hydrophobic interactions when the formed supramolecular self-assembly’s concentration was above the critical aggregation concentration. Furthermore, adjusting the ratio of β-CD_3_ to CUR, the transformation of the supramolecular self-assembled structure from spherical micelles (β-CD_3_: CUR at 2:3) to multi-compartment vesicles (β-CD_3_: CUR at 6:3) could be achieved (Figure 3B) [63]. Furthermore, in basal cell experiments, spindle-like complex micelles (β-CD_3_: CUR at 4:3) and multi-compartmental vesicles (β-CD_3_: CUR at 6:3) exhibited greater cytotoxicity, uptake capacity, and apoptosis rates than spherical complex micelles (β-CD_3_: CUR at 2:3), suggesting that altered self-assembly morphology somewhat influences the biological performance of the assemblies.

#### 2.2.2. Cross-Linked Cyclodextrin Polymer-Encapsulated Curcumin

Cross-linked CD polymers are formed by the covalent bonding of individual cyclodextrin monomers by cross-linking agents to form a cross-linked network structure, which is different from self-assembled non-covalently bonded polymerization [64]. The most common and widely reported method is the cross-linking of CD with epichlorohydrin, which was used by Chen et al. to prepare CD polymers for the encapsulation of CUR [65]. The resulting curcumin–cyclodextrin polymers exhibited higher anti-proliferative activity against A375 cells, compared to free CUR. In another study by Haimhoffer et al., polyethylene glycol was used as a cross-linking agent for CDs to form ternary complexes with CUR [66]. The resulting CD polymer effectively delivered the complexed CUR to the cell membrane, which improved the CUR permeability significantly more than the CD polymer cross-linked with encapsulation. Interestingly, the reaction of CDs with cross-linking agents such as diphenyl carbonate, diisocyanate, phthalic anhydride, and carbonyl compounds can yield cyclodextrin nano-sponges, cross-linked CD polymers with nano-sized, porous structures. Compared to common cyclodextrins, they form inclusion and non-inclusion complexes with drugs, which can improve drug delivery capacity as well as prolong the release of drug molecules. Mashaqbeh et al. prepared CD-based nano-sponges with diphenyl carbonate as a cross-linking agent, which enhanced the stability and solubility of CUR. The solubility of CUR was enhanced in the CD-based nano-sponges compared to the CUR/CD inclusion complex. In addition, the three-dimensional structure of the nano-sponges imparted higher stability to the complex [67]. In another study by Pushpalatha et al., CD-based nano-sponges (CDNS) prepared with two different cross-linking agents—diphenyl carbonate (DPC) and pyromellitic dianhydride (PMDA)—were compared for the delivery of CUR [68]. Compared to pure CUR, CUR-DPC-CDNS showed a 5-fold increase in solubility, while CUR-PMDA-CDNS showed a 16-fold increase in solubility. In cytotoxicity assays in MCF-7 cells, CUR-PMDA-CDNS exhibited higher cytotoxicity than CUR-DPC-CDNS. PMDA cross-linking may be a better method to obtain nano-sponges. In a similar study, Rafati et al. used this method to prepare CD nano-sponges, which were complexed with CUR to extend the drug release time, which was sustained over 42 h. The porous structure of the nano-sponges allowed CUR to bind to CD on the surface of the carrier and inside the cavity, exhibiting biphasic drug-release kinetics. The CUR molecules located on the surface of the nano-sponge were first released rapidly, followed by the slow release of CUR molecules located inside the cavity [69].

#### 2.2.3. Diamine-Linked CD Dimer-Encapsulated Curcumin

In contrast to the high molecular weight possessed by cross-linked CDs, γ-CD oligomers could be used as carriers of CUR for the treatment of prostate cancer cells by Harada et al. [70]. In this drug-delivery system, two γ-CDs were substituted for each CD glucopyranose unit C6A site by succinamide or urea to form a diamine-linked γ-CD dimer, which then encapsulated the CUR by hydrogen bonding. During drug delivery, the diamine linker can be hydrolyzed by intracellular enzymes, resulting in the intracellular release of the drug CUR [71,72].

**Table 2:** Summary of various cyclodextrin polymer-coated curcumin studies.

CD Type	Material Classification	Preparation Method	Characterization Techniques	Key Findings	Reference
α-CD	Self-assembled supramolecular network	-	XRD, FTIR, ^1^H NMR	The slow release of CUR is achieved by complexing with α-CD and further forming a hydrogel.	[73]
β-CD	Self-assembled supramolecular network	-	TEM, AFM, DLS, ^1^H NMR and 2D NOESY NMR	Tunable CD supramolecular self-assembled carriers were successfully constructed for the controlled release of drugs.	[63]
β-CD	Self-assembled supramolecular network	-	SEM, AFM, FTIR, XRD, UV-Vis and NMR	Amphiphilic vesicle molecules of CUR/CD were prepared for the controlled release of CUR.	[62]
β-CD	Crosslinked CD polymer	-	XRD, FTIR, DSC and UV-Vis	CUR/β-CD polymer has higher anti-proliferative activity against A375 cells compared to free CUR.	[65]
β-CD	Crosslinked CD polymer	Freeze-drying	UV-Vis, FTIR, ^1^H NMR	Epichlorohydrin and citric acid cross-linked β-CD polymers were prepared for the encapsulation of CUR.	[74]
β-CD	Crosslinked CD polymer	-	-	Elucidating the molecular mechanisms by which CUR/β-CD polymers inhibit the growth of HepG2 cells.	[75]
β-CD	Crosslinked CD polymer	Freeze-drying	DLS, ^1^H NMR and 2D NOESY NMR	A water-soluble ‘two-in-one’ polymer containing covalently bonded polyethylene glycol and βCD groups has been prepared for the encapsulation of CUR.	[66]
β-CD	Crosslinked CD polymer	-	SEM, Raman spectroscopy and DLS	Encapsulation in CDNS greatly extends the long-term photostability and anti-cancer activity of curcumin.	[20]
β-CD	Crosslinked CD polymer	-	-	CUR/CD polymers have potential in the prevention of liver injury.	[76]
β-CD	Crosslinked CD polymer	Kneading	FTIR, ^1^H NMR, TGA and UV-Vis	CUR-β-CD polymers effectively inhibited the growth of HepG2 cells, while having little effect on non-tumor cells.	[77]
β-CD	Crosslinked CD polymer (NS)	Freeze-drying	DLS, FTIR, XRD and DSC	CUR/β-CDNS prepared with dimethyl carbonate crosslinker for the encapsulation of CUR.	[78]
β-CD	Crosslinked CD polymer (NS)	Co-evaporation	DSC, TGA, FTIR, XRD, NMR. SEM and AFM	PMDA cross-linking may be a better method to obtain nano-sponges.	[68]
β-CD	Crosslinked CD polymer (NS)	Co-evaporation, Freeze-drying	XRD, FTIR, TGA, DSC and UV-Vis	The ratio of crosslinker can influence the performance of CDNS and CDNS with a proper cross-linker ratio as a promising nanocarrier.	[79]
β-CD	Crosslinked CD polymer (NS)	Co-evaporation	SEM and UV-Vis	CUR/CDNS has a stronger in vitro release than free CUR.	[80]
β-CD	Crosslinked CD polymer (NS)	Freeze-drying	FTIR, TGA, XRD, DSC and SEM	CUR/CDNS prepared with phthalic anhydride as a cross-linking agent can be used in cancer therapy.	[69]
β-CD	Crosslinked CD polymer (NS)	Freeze-drying	XRD, DSC, FTIR and SEM	Compared to the CUR-β-CD complex, CUR in cross-linked β-CDNS resulted in a more significant enhancement in drug solubility and increased the complexation stability.	[67]
β-CD	Crosslinked CD triazine polymer	Freeze-drying	FTIR and ^1^H NMR	CD polymer-coated CUR is more cytotoxic to cancer cells than free CUR.	[29]
γ-CD	Crosslinked CD polymer	Co-evaporation, Freeze-drying	IR, UV-Vis and ^1^H NMR	γ-CD polymer complexation is a promising method for improving the water solubility of CUR.	[81]
γ-CD	Diamine linked CD dimers	-	UV-Vis and ^1^H NMR	Diamine-linked γ-CD dimers can be used as novel carriers for encapsulating CUR.	[70]

### 2.3. Other Novel Cyclodextrin Nano-Supramolecular Systems with Curcumin

#### 2.3.1. Chitosan-Based Nano-Systems

**Figure 4:**
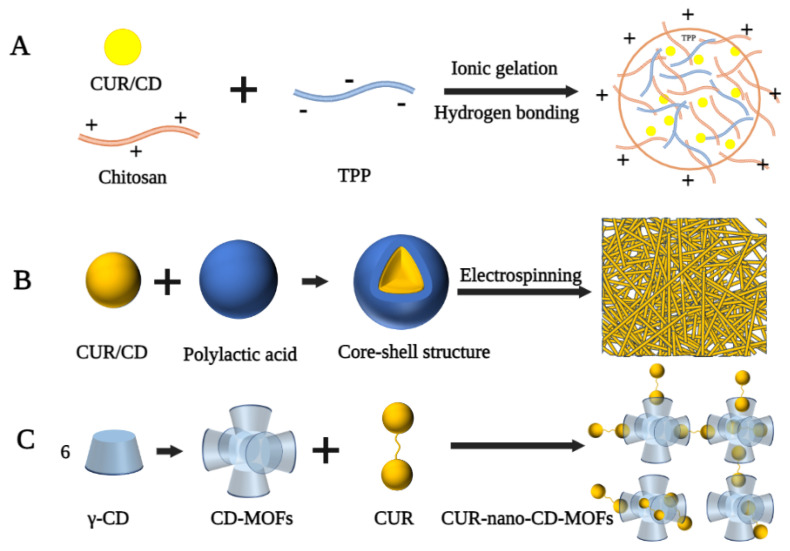
Fabrication process of different types of curcumin-loaded CD-based nanocarriers. (**A**) Chitosan-based nanoparticles [85]. (**B**) Nanofiber [88]. (**C**) Cyclodextrin-based metal–organic framework nanoparticles [89].

Chitosan (CS) is a linear polysaccharide produced by the deacetylation of chitin and is often used to develop nanomaterials as carriers [82]. In the delivery system of CUR and CD, the negative charge of CUR/CD limits its cellular delivery properties and its therapeutic efficacy. CS, as a cationic natural polysaccharide, can form more stable inclusion complexes through ionic interactions to facilitate intracellular drug transport [83,84]. Popat et al. prepared CUR/CD-CS nanoparticles with a particle size in the range of 180–200 nm, spherical shape, and zeta potential of +15 mv for the treatment of human skin cancer cells (SCC25) [85]. Highly soluble CUR/CD hollow spheres were first prepared by a spray drying method, followed by the addition of tripolyphosphate (TPP), thus encapsulating the CUR/CD using hydrogen bonding and ionic gelation of CS with TPP (Figure 4A). The encapsulated nanoparticles were still positively charged and transported CUR into cancer cells via the enhanced permeation and enhanced permeation retention effect, exhibiting higher cytotoxicity against the SCC25 cell line compared to free CUR, CUR-CS, and CUR/CD. In a similar study by Alizadeh et al., CUR/β-CD-CS and CUR/γ-CD-CS exhibited excellent in vitro release properties and high cytotoxicity against human lung cancer cells [86]. Similarly, Karpkird et al. synthesized nanocarriers consisting of CD polymers cross-linked by citric acid (pbCD) and CS for the encapsulation of CUR [87]. In vitro studies showed that the release rate of CSpbCD-CUR was slower than that of free CUR, resulting in a lower cytotoxicity of CSpbCD-CUR than pbCD-CUR or free CUR.

#### 2.3.2. Nanofibers

Electrospinning is a common method of preparing nanofibers by using an electrostatic force to stretch the electrospinning fluid [90]. Nanofibers made by this technique have many attractive properties, such as easily adjustable structure and size, large specific surface area, and diverse chemical composition, which make them suitable as transport systems for drug molecules [91]. Sun et al. prepared CUR/CD inclusion complex-loaded polyvinyl alcohol nanofibers via the electrospinning technique [92]. ^1^H NMR spectra suggested that the chemical integrity of CUR was not altered after electrostatic spinning. Therefore, the resulting nanofibers have the potential for development in drug delivery, wound healing, and cancer treatment. Rezaei et al. instead added almond gum to prepare CUR/CD inclusion complex-loaded almond gum/polyvinyl alcohol composite nanofibers [93]. The diameter of these nanofibers was in the range of 98–169 nm, and the inclusion complexes were present in a non-crystalline form. In addition, the solubility of the inclusion complex in the nanofibers was increased by 160-fold compared to that of pure CUR. In another study by Aytac et al., electrospinning was used to prepare core-shell nanofibers for the slow release of CUR [88]. In this formulation, CUR and HP-β-CD inclusion complexes were used as the core and polylactic acid (PLA) as the shell to form nanofibers with an average diameter of 695 nm (Figure 4B). In vitro release experiments showed that CUR/HP-β-CD-PLA nanofibers released CUR more slowly than CUR-PLA nanofibers during simulated gastric acid and intestinal fluid digestion due to the incorporation of a shell structure.

#### 2.3.3. Cyclodextrin-Based Metal–Organic Framework Nanoparticle

CD-based metal–organic frameworks (CD-MOFs) are practical multifunctional materials with a porous structure and good biocompatibility, which can also be used as carriers to transport drugs [94]. Chen et al. used a modified solvothermal method and PEG to prepare a nano-CD-based organic backbone for the encapsulation of CUR [89]. In this nano-system, the CD-MOFs consisted of an extended body central framework of (γ-CD)_6_ cubic units, while CUR was present in the amorphous form in the hydrophobic cavity of (γ-CD)_2_ and the cycloidal cavity of (γ-CD)_6_ (Figure 4C). The resulting CUR-Nano-CD-MOFs still exhibited high antioxidant activity compared to free CUR after 120 min of continuous UV irradiation.

Up-to-date, several cyclodextrin-based nano-supramolecular systems were also designed to encapsulate and deliver curcumin, which are summarized in Table 3.

## 3. Application of Curcumin Release from Cyclodextrin Multi-Stimulatory Response Vehicle

Among the biological activities of CUR, its anticancer properties have been a primary focus of many studies [105]. CUR is known to inhibit signaling for cancer cell growth, thereby inhibiting tumor angiogenesis and inducing tumor cell apoptosis [106]. Furthermore, CUR could benefit from targeted therapy by precise delivery. The development of smart bio-stimulatory responsive drug carriers capable of achieving precise release of CUR according to the desired environment is one of the current approaches to improve the anti-cancer effects of CUR. The CDs are also considered to be one of the most useful building blocks for constructing stimuli-responsive drug carriers [107]. Due to the self-assembly properties of CDs and dynamic reversibility based on non-covalent interactions, they can be combined with other biocompatible materials to build supramolecular polymers or nano-systems to control the precise release of CUR [108].

### 3.1. Cyclodextrin-Based Polymer Vesicles Stimulate Response to Curcumin Delivery

**Figure 5:**
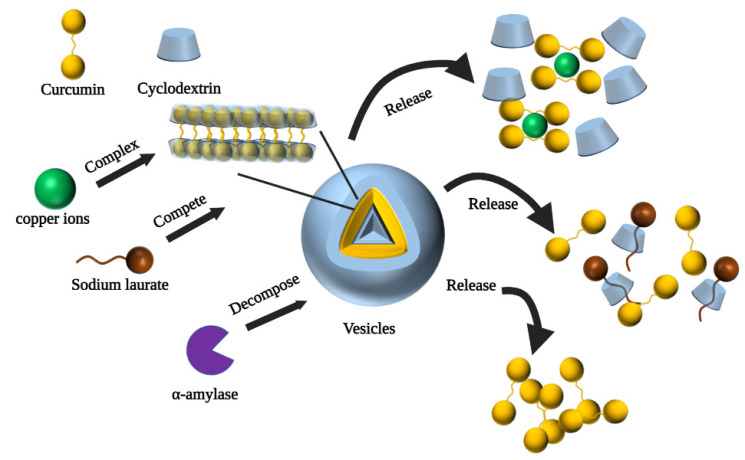
Mechanism of curcumin release from vesicles triggered by sodium laurate, α-amylase and copper ions [62].

CD-based polymeric vesicles serve as a promising drug delivery vehicle for controlled encapsulation and release of drugs, which can be designed for intelligent release in response to a range of stimuli (e.g., pH changes, enzymatic catalysis, temperature, magnetic fields, and light) [109]. Non-covalent interactions are critical in the construction of CD-based vesicles [61]. Bai et al. constructed three different morphologies of CD polymers by regulating the host–guest inclusion and hydrophilic interactions in the self-assembly system [63]. In addition, the amount of CUR released from the three different morphological carriers formed by the self-assembly at pH = 5.0 was significantly greater than that at pH = 7.4. This indicates that the release of CUR in this system can be controlled by pH and may be beneficial for cancer therapy under low pH conditions. To obtain better specificity and flexibility to adapt to the many influences changing in the organism, Ma et al. designed a CD-based multi-stimulus-responsive vesicle carrier that exhibited the ability to release CUR in response to three external stimuli [62]. Figure 5 shows the process of CUR release from sodium laurate-, α-amylase-, and copper ion-stimulated vesicles. Sodium laurate is a competing guest molecule, which replaced curcumin and bound to the CD cavity to form a new supramolecular system. Unlike this, α-amylase controlled the release of CUR by decomposing CD, i.e., disrupting the external hydrophilic layer of the vesicles, to induce the release of CUR from the vesicles. Alternatively, the addition of copper ions disrupted the hydrophobic backbone of the vesicles, and CUR formed complexes with the copper ions and detached from the vesicles.

### 3.2. Cyclodextrin-Based Nano-Systems Stimulate Response to Curcumin Delivery

In recent years, CD-based nano-systems have been widely studied in the research and development of nanotechnology [110]. Complex nano-systems constructed with CD as the basic unit can rapidly respond to microenvironmental changes and achieve the on-demand release of drugs. When designing CD-based nanodrug carriers, the primary consideration is the distinctly different physiological characteristics between human tumor cells and normal cells. For example, the tumor microenvironment pH is acidic, as contrasted with normal tissue pH, which is neutral. Therefore, drug carriers with the ability to differentiate between tumor cells and normal cells could facilitate targeted and reliable drug delivery and release [27]. Indeed, a number of pH-sensitive CD-based nanocarriers have been successfully developed as CUR carriers in the treatment of cancer [111]. Wei et al. combined a pH-responsive penetrating peptide (R6H4) with Carboxymethyl-β-CD to synthesize pH-responsive CD derivatives with cell-membrane-penetrating abilities, based on which they further formed nanoparticles with CUR and studied their pH-responsive properties [103]. The results showed that nanoparticles had higher cytotoxicity at pH 6.4 compared to pH 7.4. In addition, similar findings were obtained in cell uptake and apoptosis studies in HepG2 cells. Based on the superior physiological activity of the nanoparticles in a mildly acidic (pH = 6.4) environment, these nanoparticles demonstrated desirable anti-cancer effects in tumor-bearing mice. In another study, Aytac et al. prepared core-shell nanofibers with CUR and CD inclusion complexes as the core and polylactic acid as the shell, which exhibited pH-dependent release in 0.1 M HCl (pH 1, simulated gastric fluid). Therefore, this pH-responsive drug delivery system of core-shell nanofibers may be a promising drug carrier for targeting gastric cancer [88]. Interestingly, Wen et al. prepared γ-CD-BSA nanoparticles for the slow release of CUR by grafting γ-CD onto bovine serum albumin (BSA) using epichlorohydrin as a cross-linking agent [104]. The results showed that CUR was released in PBS (pH 7.2) for 4 h with a release rate of 57% ± 1% and in HCl (pH 1.2) for 2 h with a release rate of 15.2% ± 0.2. Therefore, in contrast to the nanoparticles obtained by Aytac et al., γ-CD-BSA nanoparticles have the potential to protect CUR in the stomach (pH 2.0) and release CUR in the intestine (pH 7.0).

In addition, certain changes in temperature and enzyme activities at the lesion site may occur. For example, because tumor cells have unlimited proliferation and high metabolism, which increases their temperature (40–42 °C) above that of normal cells (37 °C), CD-based drug carriers can achieve drug release at the lesion site through temperature changes during the targeted drug delivery phase [112]. Sedghi et al. designed a novel intelligent thermoresponsive-magnetic molecularly imprinted polymer nanocomposite for the controlled and slow release of CUR, with the ability to respond to temperature stimuli [99]. In this response system, the release of the drug could be controlled by changing the temperature because of the phase transition behavior resulting from the inclusion of N-isopropylacrylamide monomers. The drug release experiments showed that approximately 62% of the CUR was released when the temperature was at 25 °C, but about 86% of the CUR was released when the temperature was increased to 38 °C. Although this drug carrier has thermally responsive properties, it needs further improvement to be applied to human anti-cancer treatment.

In addition to pH and temperature stimulation of CD-based drug carriers for CUR release, CD-based drug carriers based on other stimuli, such as enzymes, light, and magnetic fields, are also promising for CUR release. Due to the elevated levels of enzymes such as amylase and lipase at the lesion site, different CD-based drug carriers can be designed according to the target of enzymatic degradation during the construction of the enzyme-stimulated reaction system [27]. Park et al. designed CD-coated porous silica nanoparticles, which exhibited enzyme-responsive characteristics [113]. The CD on the surface of the silica nanoparticles was hydrolyzed by α-amylase to release the drug from the porous reservoir. The ester linkage in the CD stalk was also cleaved by lipase, resulting in the release of drug molecules from the channel. Namgung et al. formed ester linkages between paclitaxel and a CD polymer using maleic anhydride [114]. Due to the presence of high levels of esterase at the tumor site, the ester linkages to paclitaxel were degraded when the drug carrier reached the tumor site, thereby achieving precise release. The constructed nano-drug carriers showed significant anti-tumor activity in a mouse tumor model. In addition, photo-stimulated host–guest interactions can be used to develop CD-based carriers for controlled drug release, which consist of guest molecules, an azo compound, and CD. Azobenzene, a compound that undergoes reversible cis-trans isomerization upon illumination with ultraviolet light, disrupted the interaction of the guest molecule with CD [115]. The negatively charged polyelectrolyte chains cannot continue to hold the drug, and therefore the drug is released [116]. However, the application of CUR as a photosensitizing active substance in photo-stimulatory reaction systems remains to be investigated. Likewise, applications regarding the multi-stimulus responsive CD-based carrier delivery of CUR remains to be developed.

## 4. Conclusions

CUR has received widespread attention due to its multiple biological activities. However, the hydrophobicity, low bioavailability, and poor chemical stability of CUR pose great challenges to its effective delivery. Combining CUR with CD to construct a supramolecular system is one of the effective strategies to improve the therapeutic potential of CUR. Many studies have demonstrated the effectiveness of CD monomers in improving the aqueous solubility of CUR. However, in the last few years, the construction of new supramolecular systems has been used to improve their capabilities in other aspects. In this review, different supramolecular systems of CUR/CD are summarized. Hydrophobic interactions between the host and guest as well as non-covalent bonding forces such as hydrogen bonding and van der Waals forces are the main driving forces in the construction of supramolecular structures of CD monomers and CUR. Moreover, the non-covalent self-assembly and the covalent polymerization of cross-linkers give the CD polymer and CUR supramolecular systems different structural features and new physicochemical properties different from those of the original constituent molecules. More importantly, some of the complex nano-systems constructed with CD as the basic unit not only improve the solubility of CUR but also release it slowly, which greatly expands the application scope of CUR.

In the field of medical research, precise drug distribution allows the drug to achieve the best biological efficacy at the lowest dose to minimize side effects. Smart stimulus-responsive drug carriers enable the release of drugs at the right time and site for precise treatment. CD-based stimulus-responsive drug carriers can drive the development of precision medicine to a certain extent. To this end, we hope that the anti-cancer activity of CUR can be maximized. We summarize and discuss different stimulus-responsive CD-based carriers for the delivery of CUR. When factors such as temperature and pH change, they cause changes in the structure of some CD-based drug carriers. It is particularly exciting to induce the release of drug CUR from these smart drug carriers and to act precisely on different cells to achieve higher therapeutic indices. Although good in vitro results have been achieved with these stimulus-responsive CD-based drug delivery carriers, their design is often complex and most of them are still in the conceptualization and validation stages. There are still many issues to be faced in the precision delivery of CUR, such as the biocompatibility and biodegradability of the system, in addition to the complex environmental changes in the human body. Overall, the application of CD-based stimulation-responsive drug carriers has advanced the development of CUR in cancer therapy, but more research is still needed to support its use in clinical treatment.

## Figures and Tables

**Table 3:** Summarized articles related to nano-systems containing cyclodextrins encapsulated with curcumin.

Types of Nanoparticles (NPs)	CD Type	The Mean Particle Size	Preparation Method	Characterization Techniques	Reference
Nanoparticles	β-CD	157 ± 38 nm	Freeze-drying	FTIR, Raman spectra, DTA, XRD, SEM	[95]
Nanoparticles	β-CD	287 ± 57 nm	-	FTIR, XRD, TEM	[96]
Nanoparticles	β-CD	-	Electrospinning	FTIR, SEM, DSC, TGA, ^1^H NMR,	[97]
Chitosan nano-particles	β-CD, γ-CD	-	-	FTIR, ^1^H NMR, UV-Vis, XRD, SEM	[86]
Chitosan nano-particles	β-CD	181 ± 45 nm	Ionic-gelation	FTIR, SEM, DSC	[87]
Nanofibers	β-CD	250–350 nm	Electrospinning	FTIR, XRD, SEM, DSC, ^1^H NMR, TGA	[92]
Nanofibers	β-CD	98–169 nm	Electrospinning	SEM, XRD, TGA, FTIR	[93]
Magneto-liposome nanoparticles	β-CD	67 nm	-	FTIR, ^1^H NMR, SEM, UV-Vis	[98]
Magnetic nanocomposites	β-CD	130–150 nm	Free radical polymerization	FTIR, XRD, TGA, SEM, VSM	[99]
Nanocapsules	β-CD	50–100 nm	-	FTIR, ^1^H NMR, UV-Vis, DSC, TEM	[100]
Nanoparticles	HP-β-CD	92–117 nm	Nanoprecipitation	^1^H NMR, DSC, TEM	[101]
Nanofibers	HP-β-CD	695 ± 280 nm	Electrospinning	^1^H NMR, CLSM, TEM, SEM, XRD, TGA	[88]
Nanofibers	HP-β-CD, HP-γ-CD	100–230 nm, 540–1340 nm	Electrospinning	FTIR, XRD, DSC, TGA, SEM, ^1^H NMR	[102]
Nanoparticles	CM-β-CD	150–200 nm	Saturated aqueous solution	FTIR, TEM, DSC, XRD, ^1^H NMR,	[103]
Nanoparticles	γ-CD	200–300 nm	Freeze-drying	FTIR, ^1^H NMR, SEM	[104]
Metal–organic framework nanoparticles	γ-CD	-	-	FTIR, SEM, TGA, DSC, XRD	[89]
Chitosan nanoparticles	HP-γ-CD	190 ± 10 nm	Freeze-drying	TEM, SEM, DLS, TGA, DSC, UV-Vis	[85]

## Data Availability

Not applicable.
